# Unusual Metabolism and Hypervariation in the Genome of a Gracilibacterium (BD1-5) from an Oil-Degrading Community

**DOI:** 10.1128/mBio.02128-19

**Published:** 2019-11-12

**Authors:** Christian M. K. Sieber, Blair G. Paul, Cindy J. Castelle, Ping Hu, Susannah G. Tringe, David L. Valentine, Gary L. Andersen, Jillian F. Banfield

**Affiliations:** a Department of Earth and Planetary Science, University of California, Berkeley, California, USA; b Department of Energy Joint Genome Institute, Walnut Creek, California, USA; c Marine Science Institute, University of California, Santa Barbara, California, USA; d Ecology Department, Climate and Ecosystem Sciences Division, Lawrence Berkeley National Laboratory, Berkeley, California, USA; e Department of Biology, St. Mary’s College of California, Moraga, California, USA; f Department of Environmental Science, Policy and Management, University of California, Berkeley, California, USA; VA Palo Alto Health Care System

**Keywords:** BD1-5, CPR, candidate phyla radiation, genomes from metagenomes, gracilibacteria, surface proteins

## Abstract

The candidate phyla radiation (CPR) comprises a large monophyletic group of bacterial lineages known almost exclusively based on genomes obtained using cultivation-independent methods. Within the CPR, *Gracilibacteria* (BD1-5) are particularly poorly understood due to undersampling and the inherent fragmented nature of available genomes. Here, we report the first closed, curated genome of a gracilibacterium from an enrichment experiment inoculated from the Gulf of Mexico and designed to investigate hydrocarbon degradation. The gracilibacterium rose in abundance after the community switched to dominance by *Colwellia*. Notably, we predict that this gracilibacterium completely lacks glycolysis, the pentose phosphate and Entner-Doudoroff pathways. It appears to acquire pyruvate, acetyl coenzyme A (acetyl-CoA), and oxaloacetate via degradation of externally derived citrate, malate, and amino acids and may use compound interconversion and oxidoreductases to generate and recycle reductive power. The initial genome assembly was fragmented in an unusual gene that is hypervariable within a repeat region. Such extreme local variation is rare but characteristic of genes that confer traits under pressure to diversify within a population. Notably, the four major repeated 9-mer nucleotide sequences all generate a proline-threonine-aspartic acid (PTD) repeat. The genome of an abundant Colwellia psychrerythraea population has a large extracellular protein that also contains the repeated PTD motif. Although we do not know the host for the BD1-5 cell, the high relative abundance of the *C. psychrerythraea* population and the shared surface protein repeat may indicate an association between these bacteria.

## Abstract (precis)

CPR bacteria are generally predicted to be symbionts due to their extensive biosynthetic deficits. Although monophyletic, they are not monolithic in terms of their lifestyles. The organism described here appears to have evolved an unusual metabolic platform not reliant on glucose or pentose sugars. Its biology appears to be centered around bacterial host-derived compounds and/or cell detritus. Amino acids likely provide building blocks for nucleic acids, peptidoglycan, and protein synthesis. We resolved an unusual repeat region that would be invisible without genome curation. The nucleotide sequence is apparently under strong diversifying selection, but the amino acid sequence is under stabilizing selection. The amino acid repeat also occurs in a surface protein of a coexisting bacterium, suggesting colocation and possibly interdependence.

## INTRODUCTION

Metagenomics data, the DNA sequences from microbial communities, can be used to reconstruct genomes from uncultivated organisms and provide insight into biological processes shaping their ecosystems. The approach has led to the discovery of numerous previously unknown phyla, many of them belonging to the candidate phyla radiation (CPR), which now appears to constitute a major part of the bacterial domain (1, 2). The candidate phylum BD1-5 was first genomically sampled from an acetate-amended aquifer (Rifle, CO) (3). The organisms were suggested to have limited metabolism and predicted to be symbionts (possibly episymbionts), but the nature of their associations with other organisms remains a mystery. Wrighton et al. (3) predicted that BD1-5 bacteria use an alternative genetic code in which the stop codon UGA encodes an amino acid. Following sampling by single-cell genomics, BD1-5 members were named *Gracilibacteria* (4). The prediction that UGA codes for glycine in *Gracilibacteria* was experimentally validated by Hanke et al. (5) through proteomic analysis of a sediment enrichment culture. However, the lack of very-high-quality genomes has limited detailed analysis of the lifestyle of *Gracilibacteria* and complicated predictions regarding the presence and absence of key metabolic pathways.

Here, we used metagenomic data from a previously performed experiment intended to simulate the Deepwater Horizon (DWH) oil spill (6) to reconstruct the first closed, circular genome (1.34 Mbp) for a *Gracilibacteria* population. The experiment was inoculated using a water sample collected from the Gulf of Mexico, and *Gracilibacteria* were detected at moderate abundance 64 days after oil droplet addition (see Materials and Methods). The genome encodes numerous proteins that could not be assigned a potential function, but genes and pathways that are present were easily recognizable. Notably, one hypervariable gene is inferred to encode a protein under strong stabilizing selection, thus likely important for survival. Even for a CPR bacterium, we note an unusual lack of core carbon compound metabolic pathways, including the complete absence of glycolysis and the pentose phosphate pathway. Glycolysis is the major pathway for sugar utilization and is present even in the very small genomes of *Buchnera* and “*Candidatus* Blochmannia,” bacteria that are obligate insect endosymbionts (7), and at least a partial pathway is present in many other symbionts. These observations raise interesting questions regarding how central carbon currencies are acquired and how reducing power is generated and recycled.

## RESULTS AND DISCUSSION

### Genome assembly and curation reveal a hypervariable gene.

The draft *Gracilibacteria* (BD1-5) genome binned by Hu et al. (6) from sample BD02T64, taken 64 days after the start of the laboratory experiment (see Materials and Methods), was selected for further curation as it comprised just 6 scaffolds. We verified that these scaffolds cluster tightly together on a tetranucleotide emergent self-organizing map (see Fig. S1 in the supplemental material), supporting their derivation from a single genome (8). Protein predictions for all of these six scaffolds required use of an alternative code in which the UGA stop codon is translated as an amino acid. Consistent with prior studies of *Gracilibacteria*, the genes were predicted using genetic code number 25 (UGA translated as glycine [9]). There have been two main ideas proposed to explain how alternative coding arises. The first relates to the low GC content of some (but not all) of the genomes it occurs in. The currently described genome fits this pattern (28.87% G+C). Alternatively, McCutcheon and Moran (10) invoke loss of peptide chain release factor 2 (encoded by *prfB*), which recognizes UGA codons, to explain the reassignment of stop UGA to tryptophan (code no. 4) in insect symbionts. Consistent with the hypothesis of McCutcheon and Moran (10), *prfB* was not detected in the genome of the gracilibacterium studied here, or in any other available BD1-5 genome. However, peptide chain release factor 1 (*prfA*) was detected, and the gene coding for it is widely identified across the CPR.

10.1128/mBio.02128-19.2 FIG S1 Emergent self-organizing map showing clear clustering of genome segments (red dots) of the scaffolds assigned to the BD1-5 bin from sample BD02T64 based on tetranucleotide frequencies. Download FIG S1, TIF file, 0.5 MB. Copyright © 2019 Sieber et al. 2019 Sieber et al. This content is distributed under the terms of the Creative Commons Attribution 4.0 International license.

**FIG 1:**
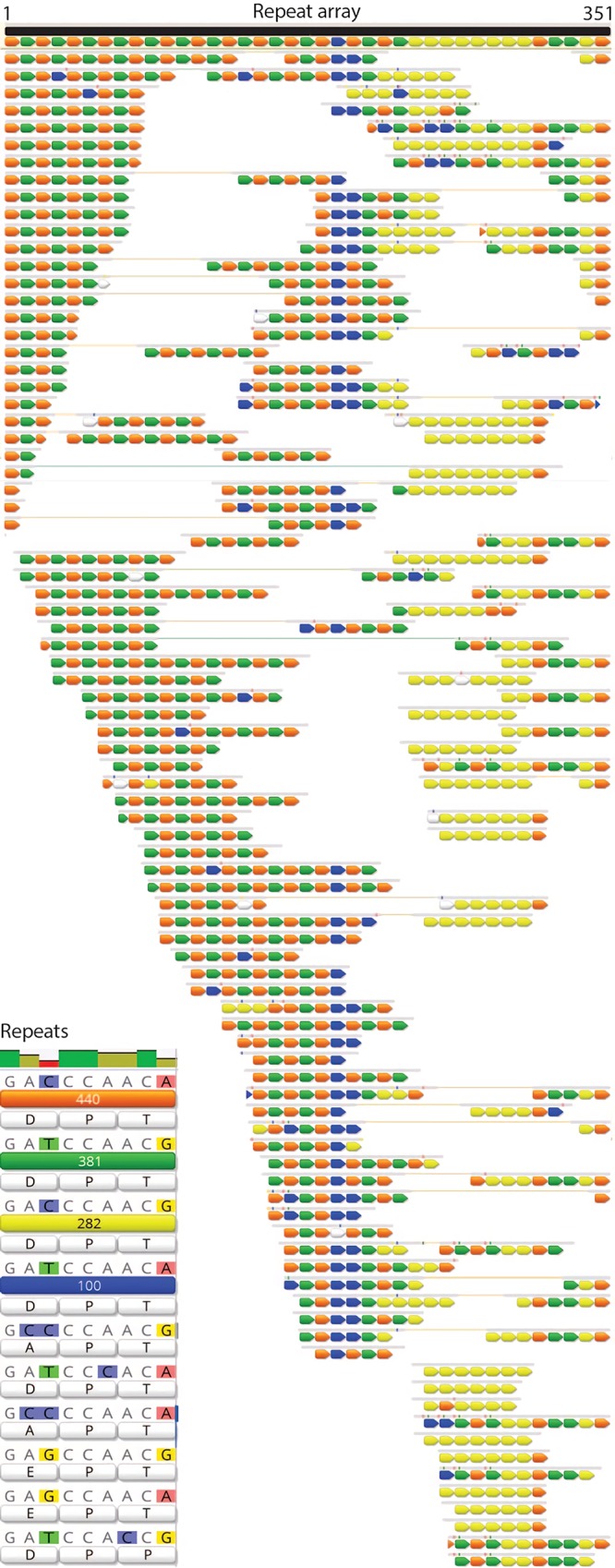
Repeat locus from the BD1-5 genome. Colored arrows represent repeated sequence blocks, the sequences for which are shown in the “Repeats” insert. Sets of arrows represent reads, and reads linked within this region to paired reads are indicated by a thin connecting line.

Prior to read-based curation, the six scaffolds were tentatively condensed into two based on perfect overlaps at scaffold ends. Local assembly errors were removed by curation, and unplaced paired reads were used to close gaps. Reads mapped to the scaffolds were visualized in Geneious (11). Notably, a region where two scaffolds were joined based on end overlaps was identified as incorrectly assembled based on the absence of perfect read support. Inaccurate read placements were associated with a hypervariable repeat locus. By manual step-by-step repositioning of paired reads (first placing reads anchored into the repeat locus boundaries, then considering paired read distances and repeat composition), it was possible to generate a representation of the sequence through the locus (Fig. 1). Due to the large size of locus compared to the paired read distance, it is impossible to determine the exact number of repeats or if the locus exhibits cell-to-cell variation in repeat number per locus. However, based on the average sequencing depth, the approximated locus is probably of about the correct length and not highly variable in terms of the number of repeated sequences.

**FIG 2:**
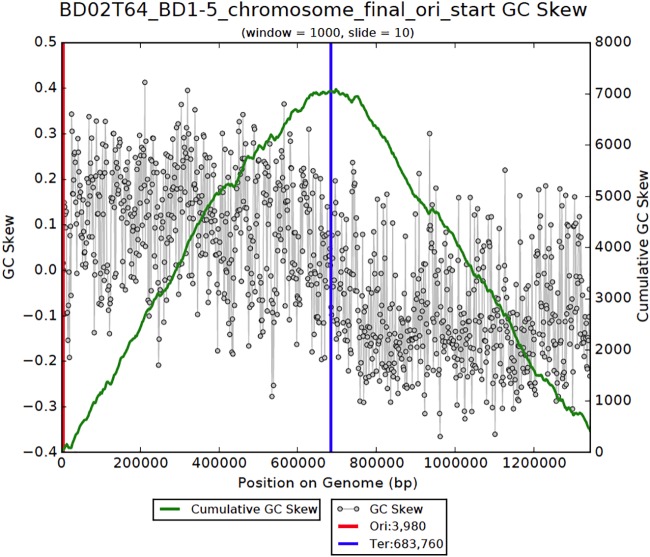
Diagram showing the GC skew (gray dots) and calculated cumulative GC skew (green line) across the finished BD1-5 genome. The pattern is typical of a correctly assembled genome of a bacterium that undergoes bidirectional replication from origin to terminus.

**FIG 3:**
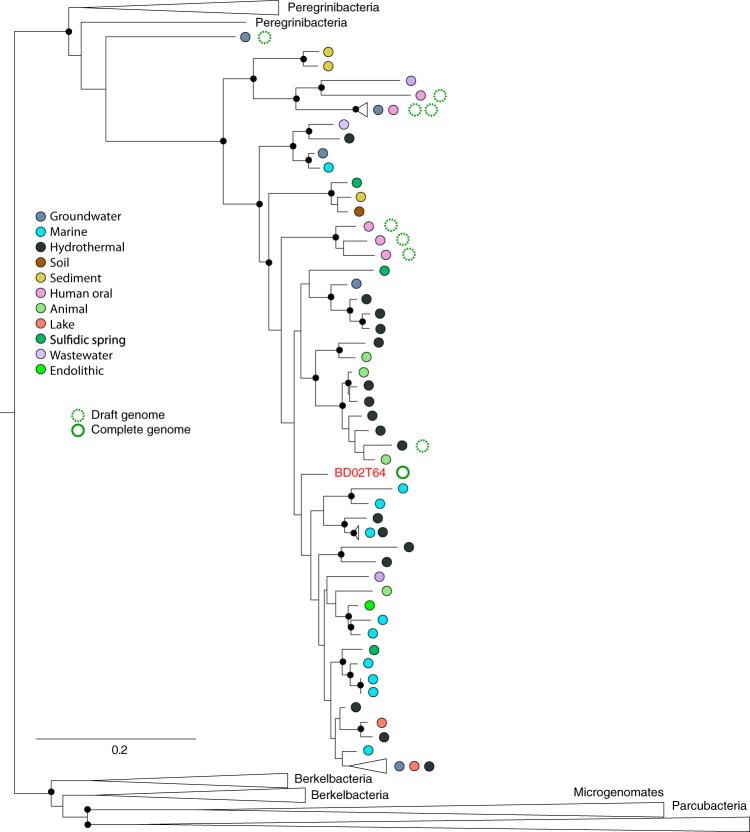
Phylogenetic placement of the *Gracilibacteria* genome from sample BD02T64 reported here. The 16S rRNA tree was constructed using the maximum likelihood method RAxML. The small black circles indicate nodes with values of >70% bootstrap support. 16S rRNA genes retrieved from genomes are indicated by green circles. Dotted circles represent published draft genomes, and the full circle indicates the finished and curated genome from this study. Colored circles indicate the type of ecosystem from which sequences were obtained. The full tree file is provided in the Data Set S1.

We verified the final genome path by calculating the cumulative GC skew of the closed chromosome sequence and identified the pattern expected for normal bacterial bidirectional replication (Fig. 2). The final assembly comprises 1.34 Mb, 1,243 protein coding genes, 33 tRNA genes and one set of rRNAs (Table 1). According to an RAxML tree based on 16S rRNA genes, the closest relative to our organism was sampled from deep sea sediments; other closely related sequences are from marine environments (Fig. 3).

**TABLE 1:** General information about the *Gracilibacteria* genome

Genome statistical parameter	Result for parameter
Genome size (Mb)	1.343
GC content (%)	28.87
Avg coverage	42.26
No. of:	
Protein-coding genes	1,243
tRNA genes	33
rRNA genes	1
Transcription factors	29
Secreted proteins (signal peptide)	66
Small secreted proteins (<300 aa)	24
Non-classically secreted proteins (no signal peptide)	104
Small non-classically secreted proteins (<300 aa)	61
Transmembrane proteins (>3 TM domains)	122
Transporters	80
ABC transporter-related proteins	11
Amino acid permeases	34

10.1128/mBio.02128-19.9 DATA SET S1 Newick version of the 16S rRNA tree with full sequence identifiers. Download Data Set S1, TXT file, 0.1 MB. Copyright © 2019 Sieber et al. 2019 Sieber et al. This content is distributed under the terms of the Creative Commons Attribution 4.0 International license.

The predicted amino acid sequence of the BD1-5 gene containing the repeat region is shown in Text S1, part A, in the supplemental material. Some repeat types occur in blocks, and some repeat types alternate, but overall the most striking feature of the locus is the high level of apparent cell-to-cell heterogeneity (Fig. 1). Variant calling in reads mapped to the full-length protein identified 17 synonymous single nucleotide substitutions and zero nonsynonymous substitutions, with the exception of instances occurring only on a single read. In fact, this gene contains the highest proportion of synonymous substitutions in any genes in the BD1-5 genome.

10.1128/mBio.02128-19.1 TEXT S1 (A) Sequence of the *Gracilibacteria* repeat protein. The sequence is approximate in terms of repeat number and read arrangement due to the limitation of the read length (see Fig. 2). Interestingly, “*Candidatus* Gracilibacteria bacterium” HOT-871 (RefSeq GCF_002761215.1) does not have this protein. (B) Curated 3,459-aa sequence of the large *Colwellia* surface protein that also contains the PDT repeat. The sequence is predicted to have two carbohydrate binding domains and a central right-handed beta helix region. The repeat region appears just after the second carbohydrate binding domain. Download Text S1, PDF file, 0.1 MB. Copyright © 2019 Sieber et al. 2019 Sieber et al. This content is distributed under the terms of the Creative Commons Attribution 4.0 International license.

Within the repeat region, the incidence of synonymous versus nonsynonymous substitutions is shown in Fig. 1 (insert). The four main repeat nucleotide sequence variants are indicated in orange, green, yellow, and blue, along with their translated sequences. Single-incidence sequences are indicated by white bars. Notably, the nucleotide sequences of the four major repeat variations all translate to the tripeptide amino acid motif PTD. Given that the *Gracilibacteria* population cells share near-identical nucleotide sequences genome-wide, except within this specific locus, we infer that the repeat-bearing protein may be under strong pressure to evolve at the nucleotide level. If cells acquire nonsynonymous substitutions in the repeat protein, they are apparently strongly selected against.

The PTD repeat motif is found in hypothetical proteins and predicted surface proteins of a few other organisms, including some that are eukaryotic sporozoite surface protein 2-like. A secondary structure prediction of the BD1-5 protein suggests only β sheet and coils, with the repeat motif in a coil region. We predict a single N-terminal transmembrane domain and extracellular localization of the remainder of the protein sequence, including the repeat region (see Fig. S2 in the supplemental material).

10.1128/mBio.02128-19.3 FIG S2 Tertiary structure prediction of BD1-5 repeat protein by I-TASSER showing variable repeat region in purple, transmembrane region in orange and noncytoplasmic region in turquoise. Download FIG S2, TIF file, 0.6 MB. Copyright © 2019 Sieber et al. 2019 Sieber et al. This content is distributed under the terms of the Creative Commons Attribution 4.0 International license.

We investigated codon usage in the repeat gene. The codons for D are GAC and GAT, with usage of 6.2:6.7 in the repeat gene, whereas the expected genome-wide incidence is GAC:GAT = 0.83:4.79. Synonymous substitutions within the repeats could cause ribosome pausing and modulate rates of protein folding (12, 13). While we considered that atypical codon use in this region might indicate selection for translational pausing, the corresponding 5′ tRNA anticodon position (G) enables a wobble pair to recruit the same tRNA to either GAC or GAT. The codons for P are coded for by CCA, CCG, CCT, and CCC, with an expected incidence of 1.3:0.13:0.89:0.13, whereas the repeat gene has an incidence of 11.82:0.25:1.23:0. Thus, there is evidence for strong selection for the CCA codon (considered further below). Inosine is the only tRNA-proline 5′ anticodon base that could accommodate all synonymous variants. The more prevalent codons, CCA and CCT would recruit two different tRNAs. Intriguingly the tRNA-proline gene carried by the gracilibacterium genome corresponds to CCA or CCG. In the third position of the tripeptide, T can be ACT, ACC, ACA, or ACG with an expected incidence of 2.1:0.41:2.4:0.36. However, within the repeat gene, ACT, ACC, ACA, and ACG occur with an incidence of 4.19:0:6.9:6.4. Again, with the exception of anticodon inosine wobble pairing, reliance on rare codons may indicate selection for translation pausing.

If it is advantageous for coexisting cells to have highly variable rates of translation, one might expect that the sequences would make maximal use of the available codons. Counter to this, we see reduced codon diversity. Thus, we considered that variation in the secondary structure of the RNA sequence in the repeat array may be selected for. In the secondary structure prediction for the repeat region, we note the periodic alternation of stems, comprising mostly Watson-Crick base pairs, and loops (see Fig. S3 in the supplemental material). Notably, the CCA codon (specifically the first C) is at the base of the bubbles and closes them, paired to G’s from either the first base of the first codon or the last base of the third codon. Stem-loops impact RNA folding, can stabilize mRNA, and provide recognition sites for RNA binding proteins. We speculate that nucleotide variation may impact the translation rate of this gene and lead to variation in the fitness of different population members.

10.1128/mBio.02128-19.4 FIG S3 Secondary structure of repeat locus. (A) Colored arrows represent repeated sequence blocks, the sequences for which are shown in the repeats insert. Sets of arrows represent reads. Substitutions are indicated with arrows and numbers. (B) Secondary structure of repeat locus. (C and D) The consequence of substitutions 1 (C) and 2 (D) on the secondary structure are indicated in local regions. Download FIG S3, TIF file, 2.3 MB. Copyright © 2019 Sieber et al. 2019 Sieber et al. This content is distributed under the terms of the Creative Commons Attribution 4.0 International license.

We searched the genomic region flanking this gene but did not identify a known mechanism for site-directed mutagenesis within the repeat locus. Genes with functions linked to DNA repair and recombination are found in close downstream proximity (*uvrC* excinuclease [5,798 bp downstream], an exodeoxyribonuclease III gene [8,336 bp downstream], and DNA recombination-mediator protein gene, *dprA* [30,798 bp downstream]). Perhaps this organism possesses a DNA mutator, which mediates targeted diversification in the repeat locus. It is unlikely that the organism is deficient in repair enzymes, as sequence variation is not elevated elsewhere in the genome. Perhaps nucleotide heterogeneity arose due to suppressed proofreading in this region, but we have no explanation for how this might have occurred.

In the current study, it is difficult to evaluate locus length variation because read lengths are short compared to the length of the repeat arrays. Locus length variation is expected, given the presence of perfect repeat arrays. In bacterial genomes, repeat regions may expand and contract due to either recombination or slipped-strand mispairing (SSM [14, 15]), resulting in population variability in terms of tripeptide motifs that may impact three-dimensional protein structure and ligand binding. The relationship between microsatellite length and point mutation has been described elsewhere and generally predicts that as a locus expands, base substitutions accumulate and suppress further SSM (16, 17). If SSM is undesirable, it is advantageous to include nucleotide variants that offset repeat pairing and thus prevent slippage.

Examination of the 5′- and 3′-untranslated regions flanking the repeat gene uncovered two sequences capable of forming stem-loops with notably long stems (14 to 15 bp) and 4- to 6-bp loops (see Fig. S4 in the supplemental material). As DNA or RNA structures, these stem-loops may play a role in recombination or as transcriptional regulation signals for the repeat-containing gene, respectively.

10.1128/mBio.02128-19.5 FIG S4 Position and structure of stem-loops located in the untranscribed regions (UTRs) of the BD1-5 repeat protein. The 3′ stem-loop is 5 bp from the gene end to 31 bp downstream (ATTAAAAAAAGAGATTCGTATATCTCTTTTTTTAAT [Δ*G* = −10.75 kcal/mol]). The 5′ stem-loop is 62 to 94 bp upstream (AAAAAACAACTCATTTTTATGAGTTGTTTTTT [Δ*G* = −12.49 kcal/mol]). A highly similar (88% identical) stem-loop sequence was identified elsewhere in the genome within the 5′ end of a putative type II/IV secretion system gene (AAAAAACACTGATAAAAATCAGTGTTTTTT; coordinates 167813 to 167842 [Δ*G* = −11.49 kcal/mol]). Download FIG S4, TIF file, 0.2 MB. Copyright © 2019 Sieber et al. 2019 Sieber et al. This content is distributed under the terms of the Creative Commons Attribution 4.0 International license.

Some reads mapped to the BD1-5 repeat region had paired reads that were not placed in that genome. Comparison of the non-repeat regions of these reads and the sequences of their unplaced paired reads to the genomes of other community members revealed 100% nucleotide matches to a region on BD02T64_scaffold_179, part of a draft Colwellia psychrerythraea genome (BD02T64_Colwellia_psychrerythraea_38_180_partial). Thus, we concluded that a region within the genome of this abundant population has the same PTD repeat as found in the *Gracilibacteria* protein (Text S1, part B). After curation of the region, the *C. psychrerythraea* protein is predicted to be 3,459 amino acids in length, with a signal peptide and extracellular localization, possible galactose/carbohydrate-binding domains, pectin lyase/virulence domains and parallel β helix repeats. The repeat occurs within a structure that otherwise consists of a mixture of α helices and β sheets but is in neither of these. Besides the repeat region, the *C. psychrerythraea* protein does not share any sequence identity (<10%) with the *Gracilibacteria* protein. Also, the TGA codon, which is repurposed in *Gracilibacteria*, is not used in either of the repeat regions.

Within the *Colwellia* protein, reads carry up to 11 repeats (see Fig. S5 in the supplemental material). As for BD1-5, it is impossible to detect variation in repeat number in each cell due to the read length limitation, but one read has only five repeats. In virtually all cases, the nucleotide repeat is encoded by a single 9-mer (yellow), This 9-mer is prominent toward the end of the *Gracilibacteria* repeat region. The loci in both genomes terminate with the same 9-mer (orange in Fig. S5). The essentially perfect repeated sequence would make this region prone to replication slippage, leading to cell-to-cell variation in the number of tripeptides in the protein.

10.1128/mBio.02128-19.6 FIG S5 Small subset of the *Colwellia* genes predicted to include a PTD repeat. Repeats code for the tripeptide PDT. The black bar indicates a deletion in one variant. The sequence of yellow and orange 9-mers is provided in Fig. 1. Download FIG S5, TIF file, 1.8 MB. Copyright © 2019 Sieber et al. 2019 Sieber et al. This content is distributed under the terms of the Creative Commons Attribution 4.0 International license.

Interestingly, several of the *Gracilibacteria* proteins encoded immediately adjacent to the variable PTD protein have the highest similarity to proteins in organisms that are not part of the CPR. One is most similar to a protein from Colwellia psychrerythraea, although the percentage of amino acid identity is low (∼53%). To rule out chimeric assembly of sequence from another bacterium in this genomic region, we confirmed the expected alternative coding throughout (and paired-read placements were verified during the main curation phase). Thus, the region encoding the *Gracilibacteria* variable repeat gene and adjacent genes may have been acquired from a bacterium related to Colwellia psychrerythraea.

### Metabolic analysis.

The biosynthetic pathways easily recognizable in the genome are for ribosome-based protein synthesis, nucleic acid synthesis and interconversion, DNA repair, peptidoglycan production, secretion, pilus production, and cell division. However, as for other members of the CPR, this gracilibacterium appears to lack the ability to synthesize lipids needed for construction of the cytoplasmic membrane (and, there is no pathway for synthesis of lipid A required for a Gram-negative cell envelope). Thus, these cells are predicted to be either symbionts or closely dependent on other community members for key building blocks. The genome lacks a CRISPR-Cas system for phage defense, but has a restriction modification system that may serve this purpose (18, 19). Absent are almost all pathways for amino acid synthesis, leading us to conclude that amino acids needed for protein biosynthesis are derived through breakdown of externally derived peptides. Many different types of peptidases and proteases are available for this process.

For nucleic acid synthesis the genome encodes the steps required to interconvert nucleotides. We also identified most of the genes required for biosynthesis of purines and pyrimidines from glutamine and aspartate; these genes are relatively uncommon in CPR. IMP can be converted to AMP, ADP, and ATP and incorporated into RNA and DNA. Enzymes were also identified to interconvert forms of GDP and GTP. Genes of the one carbon pool by the folate pathway were identified, enabling transfer of C1 groups during nucleotide metabolism, but genes for folate biosynthesis were not identified.

Perhaps the most surprising feature of this bacterium is the complete lack of genes for glycolysis and the pentose phosphate pathway, which makes this genome distinct from other *Gracilibacteria*, and possibly even from all other bacteria. At least partial pathways are present in other *Gracilibacteria*, and the first reported genomes have full pathways to convert glucose to pyruvate and fermentation-based metabolisms (3). More broadly, at least parts of these pathways are present in the most minimal CPR genomes. However, this is the first genome from a major subgroup within *Gracilibacteria* (Fig. 3), so it remains to be seen whether this is a common trait. The absence of these pathways raises two questions: (i) the nature of central carbon metabolism in these organisms and (ii) how ATP, NADH, NADPH, and ferredoxin are reduced and recycled.

Potentially addressing the first question, we identified a variety of pathways for production of central carbon currencies. We identified a putative two-subunit ATP citrate (pro-S)-lyase (EC:2.3.3.8) (genes 1051 and 1052), a complex rarely detected in CPR. This annotation (versus citrate synthase) was supported by HMM homology and the presence of the active site residues GHAGA (20). Via this complex, citrate can be converted to acetyl-CoA and oxaloacetate. Citrate may be obtained from external sources via two putative citrate transporters. Intriguingly, both ATP citrate (pro-S)-lyase subunits are most similar to predicted proteins in archaea, suggesting their acquisition via lateral gene transfer. We predict that oxaloacetate derived from breakdown of citrate is converted to pyruvate via a 2-oxoacid ferredoxin oxidoreductase (OFOR). Pyruvate can also be produced from phosphoenolpyruvate via a pyruvate kinase, and from malate (1.1.1.38) and serine (4.3.1.19). Overall, amino acids scavenged from the environment appear to feature prominently in the metabolism of this gracilibacterium, and some are converted into the nitrogen and carbon storage compound cyanophycin.

Addressing the second question, we identified many reactions that oxidize or reduce energy currencies via transformation of small carbon compounds. Specifically, pyruvate conversion to acetyl-CoA via OFOR consumes NADH while reducing ferredoxin. The ferredoxin may be reoxidized via either a cytoplasmic or membrane-bound ferredoxin reductase (FNR) that also converts NADP^+^ to NADPH. NADH may be regenerated in the production of pyruvate from serine or malate. Like citrate, malate may be obtained from external sources. Other reactions, such as those involved in peptidoglycan synthesis and interconversion of tetrahydrofolate compounds, also interconvert energy currencies. Enzymes that respond to oxidative stress response also may provide electron sinks.

**FIG 4:**
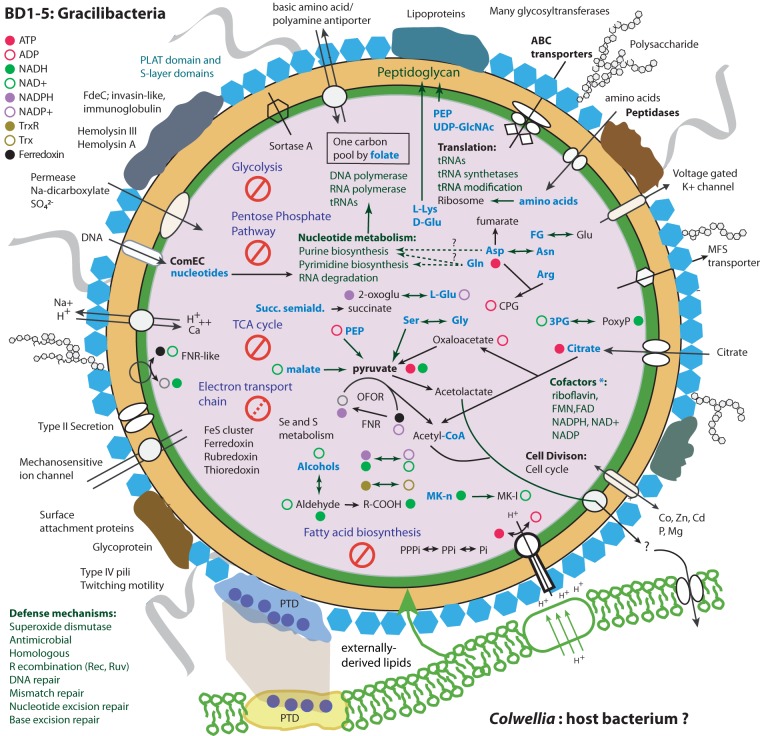
Cell cartoon depicting a reconstruction of the metabolism of the gracilibacterium. Bold text indicates prominent functions, blue text indicates resources inferred to be externally derived. * indicates that reactions for biosynthesis of cofactors require a precursor compound. Abbreviations: PEP, phosphoenolpyruvate; UDP-GlcNAc, UDP-*N*-acetyl-α-d-glucosamine; OFOR, 2-oxoacid ferredoxin oxidoreductase; 3PG, 3-phospho-d-glycerate; 3-PoxyP, 3-phosphonooxypyruvate; 2-oxoglu, 2-oxoglutarate; FNR, ferredoxin reductase; PPPi, PPi, and Pi: phosphate compounds interconverted by inorganic pyrophosphatase; Mk-n, metaquinone; Mk-l, metaquinol; Succ. semiald., succinate semialdehyde; l-Glu, l-glutamate; R-COOH, a carboxylic acid; CPG, cyanophycin; FG, *N*-formyl-l-glutamate. PTD is a tripeptide repeat.

Many CPR bacteria generate ATP via substrate-level phosphorylation reactions that produce compounds such as acetate, but genes for production of these short-chain fatty acids were not identified. ATP required for DNA and RNA biosynthesis may be formed via the F-ATP synthase complex (complex V). Given the lack of an electron transport chain that could pump protons, proton motive force (PMF) could be stolen from attached host cells if tight junctions are formed (19, 21). Such close physical associations have been reported for another CPR bacterial group, *Saccharibacteria* (TM7), which attach to host *Actinobacteria* cell surfaces (22). Alternatively, proton motive force could be generated by cytoplasmic drawdown of H^+^ via reactions involved in breakdown of amino acids and other compounds, Na^+^/H^+^ antiport, or consumption of H^+^ by superoxide dismutase. The ATP synthase may also be used reversibly to generate proton motive force (as suggested by Wrighton et al. [3]), but no complexes were identified that could make use of the generated PMF. Specifically, there is no indication of hydrogenases, which occur in some other CPR members. Lacking also are other electron transport chain components, such as NADH dehydrogenase, succinate dehydrogenase, and cytochrome *c* reductase/oxidase, and most steps of the tricarboxylic acid cycle (Fig. 4).

A variety of transporter types were predicted, presumably addressing the need to acquire compounds from other cells or detritus. There are many hypothetical membrane-associated proteins with multiple transmembrane (TM) domains that also may serve a transport role. Overall, 122 transmembrane proteins (>3 transmembrane domains) and 80 transporter proteins were identified (Table 1; see Table S1 in the supplemental material). The genome encodes an intriguing 990-aa protein predicted to contain 32 transmembrane domains (gene 860). A large-scale analysis of TM-rich proteins in the NCBI nr database revealed that very few have 32 or more TM domains, and only a few related proteins are known (mostly in other *Gracilibacteria*). The function of this enigmatic protein is uncertain as the only domain predicted is DUF2339 (hypothetical membrane protein).

10.1128/mBio.02128-19.8 TABLE S1 Prediction of transporter proteins, secreted proteins, and transmembrane proteins. Download Table S1, XLSX file, 0.1 MB. Copyright © 2019 Sieber et al. 2019 Sieber et al. This content is distributed under the terms of the Creative Commons Attribution 4.0 International license.

A notable feature of the *Gracilibacteria* genome is the prominence of secretion mechanisms and secreted proteins. We identified 66 such proteins using a combination of three methods to predict signal peptide-mediated export, of which only 24 are shorter than 300 amino acids. A further 104 proteins are predicted to be secreted via nonclassical pathways that do not use a signal peptide. Of these, 43 are larger than 300 amino acids. In addition to a sortase (typically found in Gram-positive bacteria and common in CPR), we identified genes of the type II and IV secretion pathways that are generally associated with Gram-negative bacteria, including multiple copies of SecA, -D, -F, -Y, -E, and -G). SecYEG form the central translocase across the inner membrane, SecA guides proteins to the translocase channel and is the ATPase, and SecF promotes release of the mature peptide into the periplasm. Thus, the identified components provide the functions required for secretion in non-Gram-negative bacteria. Intriguingly, 15 general secretion protein G proteins (GspG, alternatively PulG) are predicted, as well as GspE. These are large proteins, on average 528 aa in length. GspG is the major pseudopilin present in a pseudopilus, and GspE is an ATPase involved the assembly of the pseudopili. In addition, we identified around 12 type IV pilus assembly protein subunits, some in multicopy. Type IV pili allow the transfer of genetic material representing PilV, -C, -B, and -W and are involved in twitching motility (the genome also has two *pilT* genes). PilD (leader peptidase) was also identified. We did not identify PilQ, consistent with lack of outer membrane. Pili may be involved in attachment and interorganism interactions, as well as uptake of DNA. Competence genes were also identified (19, 21, 23).

From the perspective of the cell envelope, the biosynthesis pathway for peptidoglycan is complete, although the requirement for precursor UDP-*N*-acetylglucosamine from external sources is predicted. Predicted are genes to convert phosphorylated isoprenoid into a precursor for peptidoglycan, but the genome lacks the archaeal mevalonate and bacterial MEP (2-*C*-methyl-d-erythritol 4-phosphate) pathways. It has geranylgeranyl diphosphate synthase, but the reason is unclear. In addition, we identified three genes that degrade l-lysine and d-glutamate that may feed intermediates into two different steps within the peptidoglycan biosynthesis pathway. The genome contains many genes for polysaccharide synthesis (e.g., no. 444-460) and for proteins with S-layer domains. Thus, we anticipate a cell-wall-containing peptidoglycan with a periodic surface layer, many and potentially diverse pili, and a variety of large extracellular proteins and polymeric substances (Fig. 4). Interestingly, some S-layer proteins may have toxin domains (e.g., 1226, predicted to have polycystin-1, lipoxygenase, and alpha-toxin domains). Other large proteins have annotations suggestive of hostile interactions with other organisms (e.g., insecticidal toxin complex protein [TccC]), and there is a predicted invasin domain in one large protein in the genome.

In terms of the ability to respond to environmental conditions, the genome encodes at least four RelA/SpoT domain proteins, three of them encoded sequentially and one larger multidomain protein encoded elsewhere. These may function in response to nutrient limitation. Also identified are two 8-oxo-dGTP diphosphatase genes to prevent misincorporation of the oxidized purine nucleoside triphosphates into DNA and proteins with antioxidant functions, including superoxide reductase and enzymes to reduce oxidized methionine.

We conclude that the inferred putative symbiotic lifestyle of *Gracilibacteria* differs in notable ways from those of other obligate host-associated organisms. The genome size is large, compared to those of most obligate host-associated organisms (usually <1 Mbp in length [24]). Host-associated bacteria that have experienced moderate genome reduction retain genes for synthesis of fatty acids and peptidoglycan (but not for lipopolysaccharide [LPS] or phospholipids), whereas those that have undergone extreme genome reduction have essentially no genes for cell envelope biosynthesis (10). In contrast, the gracilibacterium seems to rely entirely on externally derived fatty acids. It retains genes for regulation of gene expression (e.g., two-component systems and various transcriptional regulators), DNA repair, and homologous recombination, whereas these genes are often lost in symbionts (7). Overall, the genomic features of this gracilibacterium only overlap partially with those of host-associated bacteria, which have experienced rapid genome decay.

### Conclusions.

Among the most intriguing aspects of the *Gracilibacteria* genome studied here is the variable nucleotide locus that encodes a conserved tandem PTD tripeptide repeat protein. The gene appears to be under selective pressure to preserve this sequence, as nucleotide variation is localized to this repeat locus almost exclusively as synonymous codons. We infer that the protein has a function strongly tied to the fitness of this organism. The PTD repeat sequence also occurs in coexisting *Colwellia* that became abundant late in the experiment (6) when the gracilibacterium was detected (see Fig. S6 in the supplemental material). It is unlikely that co-occurrence of the repeat is a coincidence, as this sequence is relatively uncommon, even in public databases. However, we cannot provide a definitive explanation for the shared amino acid repeat sequence in both genomes. If horizontal gene transfer was involved, only the repeat part was transferred, as the remaining sequences do not show any sequence identity. Therefore, we consider it at least equally likely that this phenomenon resulted from convergent evolution, probably with selection for an amino acid sequence with certain adhesion properties.

10.1128/mBio.02128-19.7 FIG S6 Taxonomic composition of oil spill simulation samples (6) based on relative abundance of ribosomal protein S3 genes. Abundance of *Colwellia* with repeat protein is indicated by stars. Abundance of *Gracilibacteria* is shown in red. Download FIG S6, TIF file, 1.6 MB. Copyright © 2019 Sieber et al. 2019 Sieber et al. This content is distributed under the terms of the Creative Commons Attribution 4.0 International license.

Given this, and its likely function as an extracellular protein potentially involved in attachment, we speculate that (case A) the same repeat sequence in two cell surface proteins should adhere to the same substrate (which seems very reasonable, given that adhesion is mediated by the properties of the amino acid sequences) or (case B) the proteins would adhere to each other at the repeat interface, where molecular Velcro-like binding may occur, as has been shown for other self-associating proteins (25–27). This could result in close proximity in case A or direct cell surface adhesion in case B. As case A seems highly probable based on chemical arguments and case B is less easy to establish, we favored case A in the cell cartoon in Fig. 4. However, this interaction remains speculative and requires enrichment experiments targeting *Colwellia* to determine if cocultivation with *Gracilibacteria* can be achieved.

The gracilibacterium studied here is also fascinating in terms of its unusual metabolic platform. Based on its predicted gene inventory, it is inferred to adopt the lifestyle of a scavenger or symbiont of some type (possibly as a parasite). Certainly, it requires an external source of building blocks, including lipids, amino acids, citrate, and malate. In the enrichment experiment designed to simulate the Deepwater Horizon oil spill, glucose-based compounds are not expected to be in high abundance, nor are amino acids. There is no indication that the gracilibacterium can metabolize complex oil-derived compounds. Thus, we predict that the relevant resources are probably bacterial compounds released by cell lysis (e.g., amino acids, small organic molecules, lipids, and cofactors or cofactor precursors) and those that leak from cells of coexisting oil-degrading bacteria (e.g., alcohols and aldehydes). These resources may be processed by this gracilibacterium and the by-products excreted, providing the associated organisms with compounds such as acetyl-CoA, fumarate, succinate, or acetolactate. Based on its inferred lifestyle and its phylogenetic placement within a major distinct clade (Fig. 3), we propose the name *Gracilibacteria* (phylum), *Gracilibacter* (class), *Detritibacteriales* (order), *Detritibacteriaceae* (family), *Detritibacteria* (genus) *gulfii* (species), reflecting its likely dependence on detritus and enrichment in a sample simulating the Gulf oil spill.

## MATERIALS AND METHODS

### Genome assembly and annotation.

The original study of Hu et al. (6) involved seawater samples collected from a depth of 1,100 to 1,200 m in the Gulf of Mexico in 2014. The sample derived from a region impacted by the Deepwater Horizon oil spill in 2010, but there were no oil-spill-derived hydrocarbons detected at the time of sampling. However, hydrocarbon seeps occur naturally in the general area. The *in situ* cell density was estimated at ∼5.0e + 5 cells/ml. A volume of 630 liters was returned to the surface and amended with unweathered Macondo oil (MASS oil 072610-03) at a concentration of 0.2 ppm to sustain microbial activity and maintained in the dark at 5°C while the sample was transported to the laboratory. In the experiment described previously, samples were incubated for up to 64 days in 2-liter bottles at 4°C in the dark at 0.75 rpm on a rotation carousel system. Macondo crude oil was added to the seawater in 10-μm droplets to final concentrations of 2 ppm and 0.02 ppm Corexit EC9500A dispersant (Nalco). Replicate oil-amended bottles were destructively sampled at 6, 18, and 64 days of incubation for metagenomics.

The methods for the metagenomic assembly of the genome of the BD1-5 described here, as well as the draft *Colwellia* genome, are reported by Hu et al. (6). In the current study, genome curation was conducted in Geneious (11). Curation involved visualization and validation of paired-read placements throughout. Local assembly errors were identified as regions lacking perfect read support. Gaps were inserted in these regions, and unplaced paired reads used to fill the gaps. In repeat regions, some reads were improperly placed and paired reads were missing. Curation of these regions was similar to that for local assembly errors, except reads had to be relocated manually to achieve the most parsimonious path. The same approach was used to curate the *Colwellia* genomic region that shared the same repeat sequences. After completion, the assembly was checked for repeats longer than the paired-read distance using a GC skew and cumulative GC skew calculated by previously published methods (28).

Genes of the curated, circularized BD1-5 genome were repredicted using Prodigal (29) with genetic code 25 (-g 25). Functional annotations were done using the ggKbase annotation pipeline (http://ggkbase.berkeley.edu), which searches homologs of predicted genes in the databases of KEGG (30), UniRef (31), and UniProt (32) using USEARCH (33). Amino acid sequences of genes without a significant hit were further annotated using HHblits (34) and the UniProt20 (32) database. In addition, individual genes were interrogated using HHMer (35), HHpred (36), Interproscan (37), Swiss Model (38), and blastp domain analysis. Transmembrane proteins were identified by TMHMM (39). We predicted secreted proteins using psortB (40), signalP (39), and PrediSi (41) with Gram-negative and Gram-positive prediction models, respectively. From the six predictions, we selected proteins that were identified as secreted proteins by at least three different predictions (coming from at least two independent methods). We applied SecretomeP (42) to predict nonclassically secreted proteins without signal peptide. Additionally, we removed proteins with more than one transmembrane domain predicted by TMHMM (43). We predicted transporters with TrSSP (51) and selected proteins with at least four transmembrane domains from the resulting set.

RNA secondary structure within the repeat locus was determined using YASPIN (44), and DNA secondary structure was predicted using MFold (45) for putative stem-loops flanking the BD1-5 repeat gene. Tertiary structure prediction of the BD1-5 repeat protein was performed using I-TASSER (46).

### Phylogenetic tree.

16S rRNA gene sequences were aligned using SSU-align (47) and trimmed manually. We calculated the phylogenetic tree using the maximum likelihood algorithm RAxML (48) on the CIPRES (49) web server in choosing the GTRGAMMA model and autoMRE to automatically determine the number of bootstraps.

### Nucleotide variation and codon usage analysis.

We determined single nucleotide variants using VarScan (50), with the following parameters: c10, q30, and fr0.05. This set of nucleotide variants were then assessed to determine nonsynonymous versus synonymous substitutions within each coding region of the BD1-5 genome. For each gene, we determined the number of codon positions corresponding to an amino acid substitution based on genetic code no. 25 (*Gracilibacteria*), versus those resulting in no amino acid change, counted as either nonsynonymous or synonymous, respectively. A codon usage profile was generated in Python (v.2.7.3) using the Biopython SeqUtils package. Synonymous codon usage was assessed in the repeat-rich gene for comparison with the average codon usage of all genes in the *Gracilibacteria* genome. Synonymous codons were then compared with predicted tRNA gene anticodons to address potential 5′ anticodon wobble pairing.

### Taxonomic composition of oil spill samples.

We estimated the relative abundance of taxa in the samples of the oil spill simulation of Hu et al. (6), in mapping reads to contigs with a ribosomal protein S3 gene on them. Annotation of ribosomal proteins and taxonomic classification of contigs were done using using ggKbase (http://ggkbase.berkeley.edu).

### Data availability.

The genome, with functional annotation, can be accessed at https://ggkbase.berkeley.edu/BD02T64/organisms/60439. The genome sequence has been deposited in GenBank under accession no. CP042461.
