# Supplementary material for: Unusual Metabolism and Hypervariation in the Genome of a Gracilibacterium (BD1-5) from an Oil-Degrading Community
Source: mBio. 2019 Nov 12;10(6):e02128-19. doi: 10.1128/mBio.02128-19 (PMC6851277; doi:10.1128/mBio.02128-19)
Supplement: TEXT S1 [file mBio.02128-19-s0001.pdf]

A.

>BD02T64_BD-5_variable_repeat_protein
MLNDWINDFMIFFEYHKTIVLLGFVGVLISGGVVFAFIPSFQESEKLIVI
EVSSPDQQEETDTSSEGPEGTSNETSTQASEGEGINEEVPYASAPDQSNT
SEGSENTNSTSTSNKGSSSSQNTNGSTEPDSSTVTEKGKTDIIEDSTDIV
EEVVKETEIITEEEPLQETTTPVIVTPTDPTDPTDPTDPTDPTDPTDPTD
PTDPTDPTDPTDPTDPTDPTDPTDPTDPTDPTDPTDPTDPTDPTDPTDPT
DPTDPTDPTDPTDPTDPTDPTDPTDPTDPTDPTDPTDPTDPTDPTDPTDP
TDPTDPTDPTDPTDPTDPTDPTDPTDPTDPTDPTDPTDPTDPTDPTDPTD
PLGYFNNTAYVSGGKQLKVVVDSMRSPIFPTIQACPIGFNEFKIYSNISS
ITTLELPEATWNLFISNETSGGDYYKVFNVAGVKIGAVAYKTTDPVYNYF
WAFTICLEN

B.

>BD02T64_large_Colwellia_surface_protein_with_curated_sequence
MGMPAGKNCSGKNCASARFTPEELAQGAYQGQTWNFDYAGQQYDIPPYFY
STHSPAMVALLKEQVNVLMGGSPEQTVGFQLGGNVDAIMFDSQTSSAKSL
YWGGDFSKPSLAAFNQYLQSVYQELDKEKNALPVDISGLNTIDAGHVKDN
ETVNGSNNNDLIIIKGMSQNGGSISAKNGDDVIYIADWGNAQNRSITGDS
GSNTLLLNKAIVKNVTSQGTGAGKVTFKAGGSANFNNIERVFQVDVNEDG
SLPTEFTGVKLFNELLARHIENFQIESFSEFNYAEFLLALGYNDEQVTDA
VYLNKTIDIPLYDFFKQFHVKAHSELLQNLKAHANAFKDANGNSVPDVDI
SSSGKLVYPISGHSPDAVDFYSQEEQGDAQNIKLLYKIAEAQGKALTFNQ
GPPDWIAMAEQKAVNRPRALMAQAYANGANYVVPVKNDEYLSNPGDFEFI
TDFISNNEALFDNYSPLSTIKVLHSYEAMEAAKAHRIGGEMPALFMVDEL
LEQNNIQYDLLSFSSDMWQGDPSARDIATAGVIITTTDIGYLTAEQKKLL
EPHQQKVVHIESEQDMAELFAKIGENGYVNAGEHNSDVELVVRKSNDRKA
PIVIHALNKTLNQASDEITQLNDIKVQIRYRELLKTPISKAVYYSVDDDP
STPQCIELALEETSSGVKEFTLPNLATWGVIVLEPRRGTSVNCNSYHATE
IAPLPPQDVDEGINDDVNLIINGYFTDGTADWRKVGAQLSLVHTRDIGSY
DGDEETWVGRVYQRGATGSSARAQVEFTAGSQYQASVLAKFSGDISELKL
GVRYPQADNSSKFVYPELTIESLDNGWVELSTSFTIPNELGSEGYQELYL
LTANSTEDIYFDKFTLVDTTPVSLISDVDSFINDTEADFWTGHYLNGVSL
DVMRLPLSDHLGRDSQDGKASYDNVQSWLNDNAEQGWRFATVEEMHAIAK
FYQDDTQGFHLLNGSAWSPAGEFWNANIPTSAQNDGDQMPAYSFKVTTSN
VGSVEYKPRYNVSALGASSLIVRNTPIDEIDVYTGGVSIITETDSYQNNK
ENAYWTGHNINGLNLDVMRFTVSDFVDGTNNVSFDQVNNWLAENSDWRWA
TKIEMASVVSFFANDTASFLSFNDELWSPQGSQWVGLIPGEKQDDNDTDG
VAWGFIASENNIGVVAHRHNWNSSSVTGAPLLVRESVYVSGGGTDTGEPT
AGDAIVLHVSTQSNGNSDGSVDSPYGGLLAAQQAVRSLIARGKNTLGITV
ELQSGDYFIEQGLTFTAADSGTEDAPVVYKAADGAVVNFFGGKVLNPSDF
ISANTEFTSLLIDAGVGSNIVEIDLEQAGIENLAQALGEMAPHGWNIEPS
NRVPAAMLYSGNEKMALARWPNVDEQSQYLDDKSAAHDITGMVSYTSVVD
KGLTQQDANKFSNEFNNGGGTMAVEFNRPSLWHNLSEVHLDGILAYSWEW
TYNQVKAFDADTKQLTLKRGELSGIGTNKGSHFYFENIAEELDQAGEFYI
DRDLGKLYFYPTDDFASNTTVLSTLSEPMVSISGAKHISFDGITFDTGRN
LAVKVTQGQHIAISNCTIRNFSLGGVLLDGSDNSVSGCEIANVGGYGVKV
AGGTEAKLSKSSGSPVLDTLQQPVEITAANNLVTENTIYNFAWDQKSQIP
GVSLTGVGNSATYNEIHTAPHFGILMRNTTDNVVAYNYIHDLPNYHKSDG
GALYVGIGASPHLRGNEIRNNYFEFIPTNGVYLDNFSSGLKVEHNVFNEV
GNNNDTFSGININGGGQNEMRQNFFYNAERSIKYNKFAANSLFSNYYSKM
VGVQKAFNDADVNTTPYVKYTDFIEFLAYSTEDEFHYQSSSAFENFSFNN
LVITDDRAEVTGVVEPDDKRWTVEDNALVEELTTELQTEYDVLAPIFNVR
NDKDNWRSTVESVTDQLSTSIPLMLAQVELPTEPKAESRYSDGASWLNDD
ADDYWTALNIANNGLDIMRLPLSDDLGAVGANNDQVQTWLASDDNTDNEQ

GYKWRYASEAEMIAIATFYENDGSAFLALNHAWSPHTTEQWSALIPTTQQ
GDCTHNDGCMYPSLGFTAKPNQVSNVGYKQSFNVSAKGGAALIVRDHPSE
DVGEPVADNLIVNGDFEAGTTEPWSINQGGASMALETVDGNVVAVIQNRG
GSHASTVKQSFTGLTAGETYQLSAQLNMVEVGYELGVFIGYRNEGATKDV
ALQVGSYLTTTGDWLSFDAEFTLPLDALADKGYRIWVRSTKGEHATSDPV
PSIYLDDIILVANTVDPTDPTDPTDPTDPTDPTDPTDPTDPTDPTDPTDP
TDPTDPTEPDPSQQSLLSDITSFVDDAAQPFWSAFYIEGKALDIMRLPTS
DDLDGYVNISTADTTAWLASEANIEGWRWATTAEMAAFVTFFEGDAQAFL
AMNGNVWSPKTSRDEWNALIPGEVLDESDTAGVSWGVNVLVDSIGEVVHK
HSFNTNAKGAAPLLVKTSQNAPAPDTINVAINGNLESGTEGYTLQKSSFE
VIFDAERNSNVGHVFNRTVVSSSARLAINLESGKHYTISVEAKLVDENLT
AVVGDLDLIVLYFVDGQRTSVEIDTEVLADGWITLSGEISMPEGAEITGA
KVWVKSDTINSILFDNLTVETEVVDTTNPDPEPEPDAGVADGDSEGYGIA
TTQCQADFSQLKPDALYVSANTENGDGSEASPFNSLTAALSSVAVEGVIN
QPGLTIYLREGQFHETIDSSVVTSLAGSIENPIVIEAYPCETVSFNGTTN
IESLAAINDEGEKSQWQQHYGNIFKREVTAPVWQLFVDDKQRMIARWPNA
SFDLPSATNHQSIYSDHVWAEGTLDSSKTRNGIMINDPSYHDLASAKDYR
GEPLNVDGAIVLANTASFYTYSRMVNAYDNMSSGFTSLFKTMPDDTSVGH
VAGSNTFVHEPIKSGYKGKQLSYYLEAKLELLDADNEWHYQVEDTKHYVY
LWSNSVNGPTGDVTVRDRGYAFNVSNWENVEINGLDYFASTIKCSPCNDI
TIADSNFKHSATSRRVLKQYGDDKSGYGRNQLIELYSATDKRINSGITFK
NNTVTDSDVQSLIIQGGQSTIENNLFERLDASSADTISPHATVMITKMTN
QKATFSRNTMDIIGNSTMFVPEGGSLHALYNNMITGGFAQNDGASFQLRK
AQQAAANIAYNWAHDSEKYGIRFDAPFDTTNETSGGQFGLIHHNVVWNAK
GIMVKGDDHRVYHNTTWNNADIDQRMLVDPNPYYRHERTVTSNNASDSIS
QDRLAESESLAGVVGNNFNGLKESVSLVSQLTDMFNYDFRPLREGALHNS
GAIIDSPNSYAAYIGDAPLPQYEEATVQGSTLDLGAYELDAPYYWIPGYK
TAKASFAIPRNELIDVAAKPVSGNVSIMWRDGYQALSHRLYLASTKAEIE
AITLGDIDSDLYKGNFVAGEANIYESTTTLAPGSYYWRVDAVNIDGSVIQ
GDTWKFIVE
