# Supplementary figures and images for: Unusual Metabolism and Hypervariation in the Genome of a Gracilibacterium (BD1-5) from an Oil-Degrading Community
Source: mBio. 2019 Nov 12;10(6):e02128-19. doi: 10.1128/mBio.02128-19 (PMC6851277; doi:10.1128/mBio.02128-19)

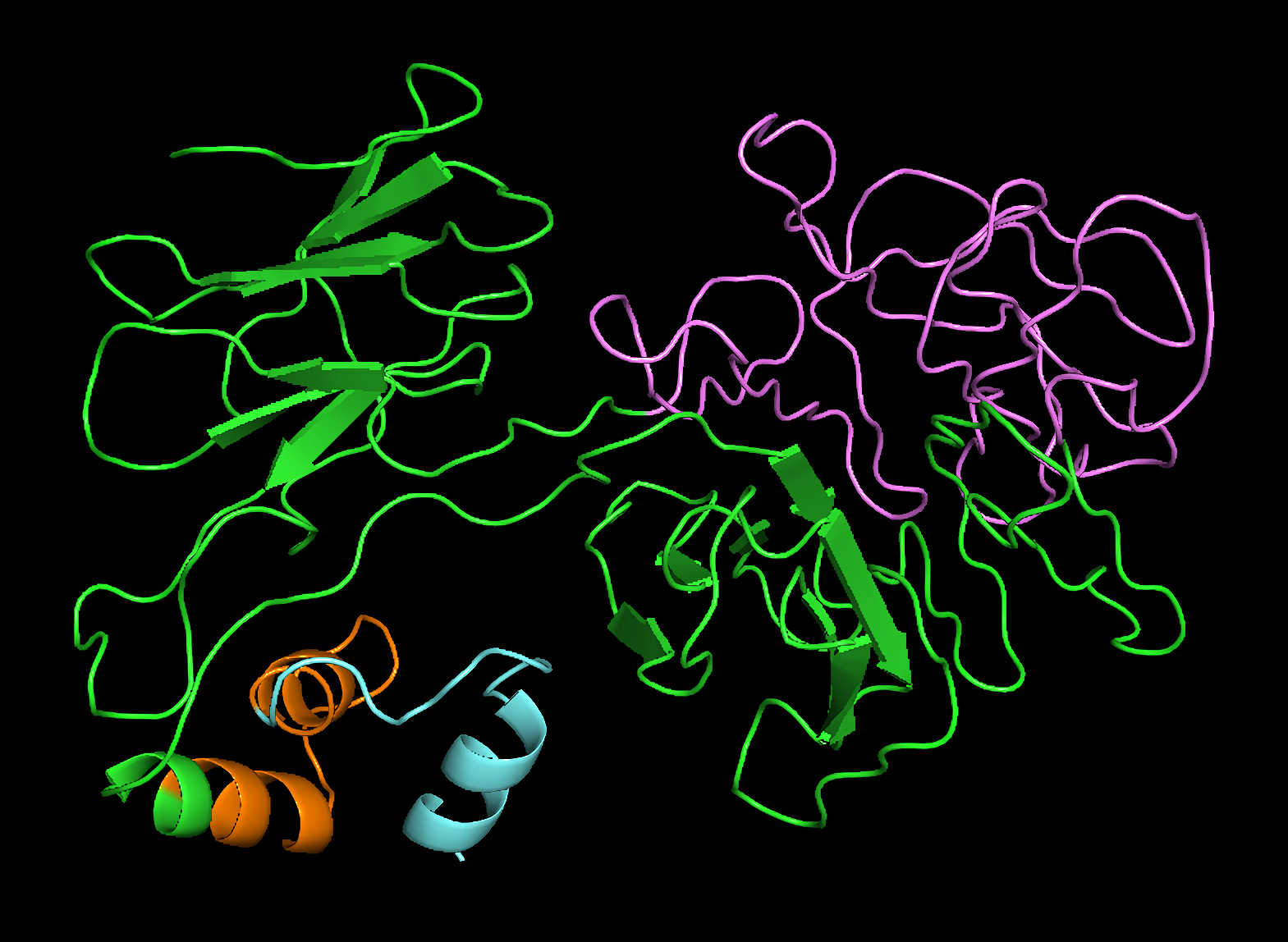

Supplement: FIG S2 [file mBio.02128-19-sf002.tif]

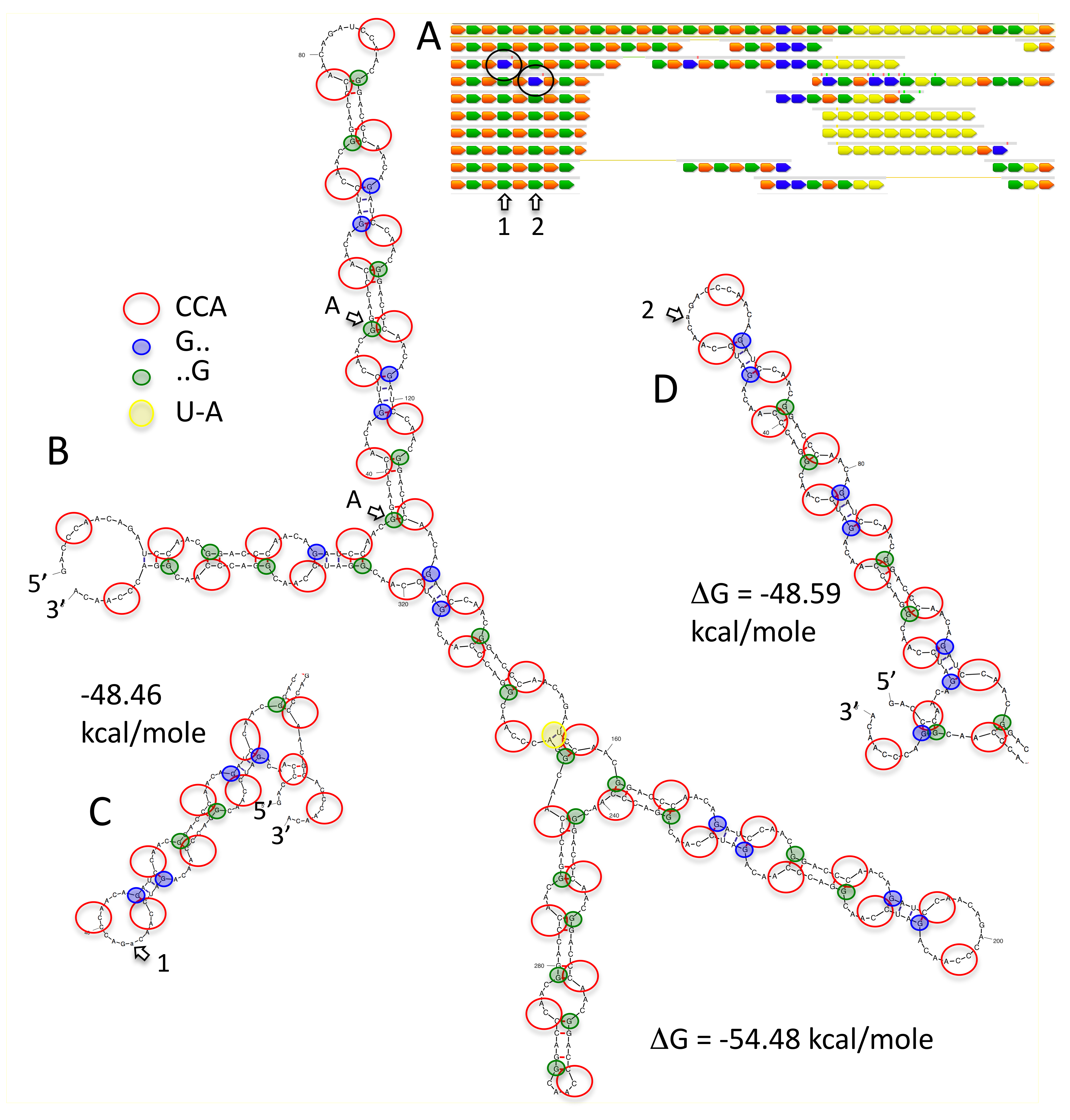

Supplement: FIG S3 [file mBio.02128-19-sf003.tif]

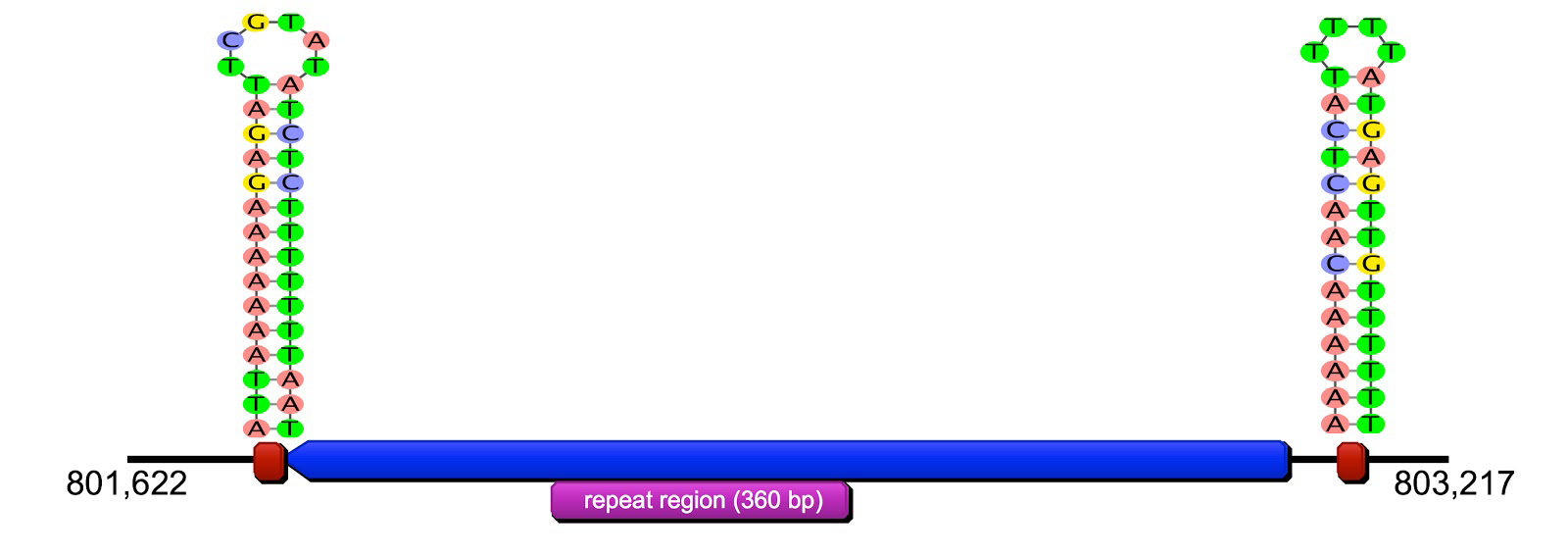

Supplement: FIG S4 [file mBio.02128-19-sf004.tif]

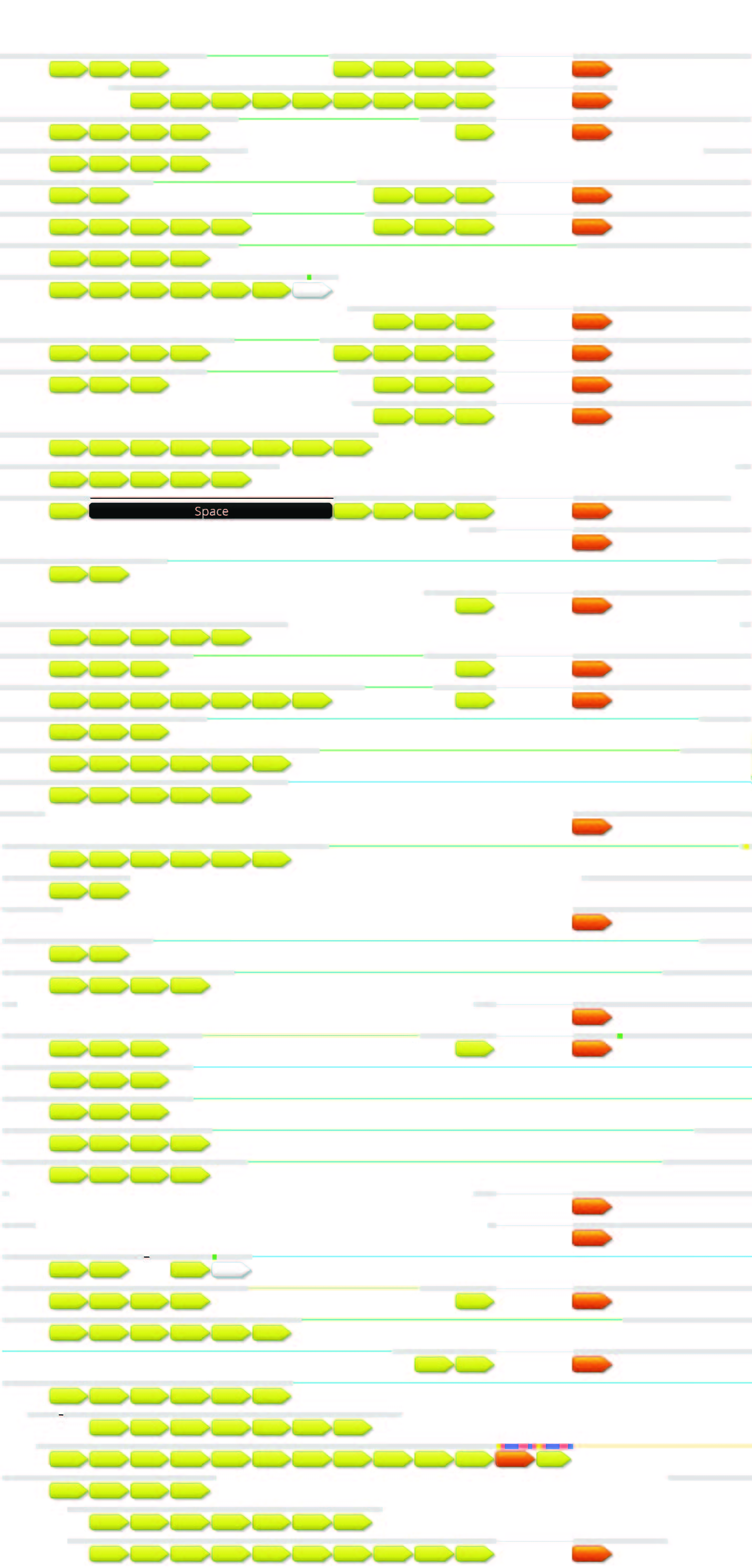

Supplement: FIG S5 [file mBio.02128-19-sf005.tif]

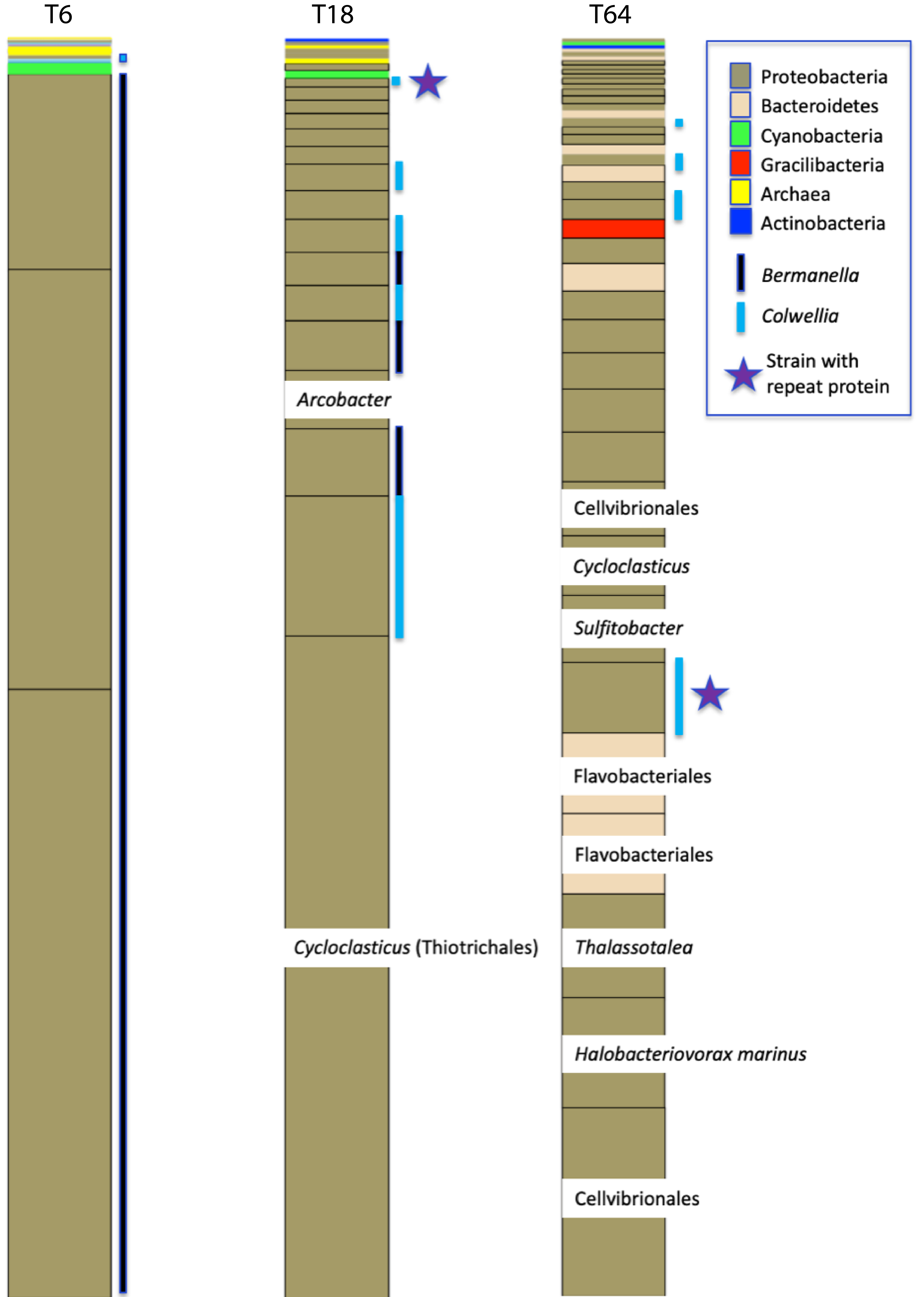

Supplement: FIG S6 [file mBio.02128-19-sf006.tif]
